# Adverse Allergic Reaction to Intrathecally Administered Technetium-99m Diethylenetriamine Pentaacetate: A Case Report

**DOI:** 10.1055/s-0043-1771281

**Published:** 2023-09-06

**Authors:** Ahalya Nair, Guneshwaran Poolpandian, Kabilash Dhayalan, Jayaram Saibaba

**Affiliations:** 1 Department of Nuclear Medicine, Jawaharlal Institute of Postgraduate Medical Education and Research (JIPMER), Puducherry, India; 2 Department of Anesthesiology and Critical Care, Jawaharlal Institute of Postgraduate Medical Education and Research (JIPMER), Puducherry, India; 3 Department of Neurology, Jawaharlal Institute of Postgraduate Medical Education and Research (JIPMER), Puducherry, India

**Keywords:** adverse allergic reaction, CSF rhinorrhea, intrathecal route, radionuclide cisternography, ^99m^ Tc DTPA

## Abstract

Intrathecal administration of radiopharmaceuticals is an infrequently performed procedure in most nuclear medicine facilities. It is possible that adverse allergic reactions following the intrathecal administration of radiopharmaceuticals often go unreported. Here we present a case of spontaneous cerebrospinal fluid rhinorrhea with recurrent bacterial meningitis, who underwent radionuclide cisternography for localization of the site of leak and developed an adverse allergic reaction following the intrathecal administration of technetium-99m diethylenetriamine pentaacetate that resolved with appropriate treatment. Imaging, however, could be carried out to our satisfaction and the allergic reaction did not interfere with, or result in discontinuation of the scan procedure.

## Introduction

The accurate preoperative localization of a cerebrospinal fluid (CSF) leak is an essential prerequisite to successful surgical repair. This is especially important in cases where recurrence has occurred following a blind epidural blood patch repair or where conservative management is unfavorable due to the development of complications such as bacterial meningitis. Various imaging modalities are available to achieve this end. Computed tomographic (CT) cisternography with metrizamide contrast is considered the gold standard for this indication. However, it has a variable detection rate between 40 and 92%. 1 2 Other modalities such as a combination of high-resolution CT and magnetic resonance cisternography and radionuclide cisternography are seldom performed. Radionuclide cisternography using technetium-99m diethylenetriamine pentaacetate ( ^99m^ Tc DTPA) lends itself as a useful procedure with a high sensitivity for the detection and localization of the leak site, especially in cases with intermittent symptoms. 3 4 However, caution must be exercised as the intrathecal route of administration of a radiopharmaceutical may result in idiosyncratic reactions requiring urgent care and further exploration. 5 6 Herein, we present an interesting case of spontaneous recurrent CSF rhinorrhea with a history of failed patch repair where intrathecal administration of ^99m^ Tc DTPA was followed by an immediate allergic reaction to one or more of the components of the radiopharmaceutical preparation.

## Case Presentation

**Fig. 1:**
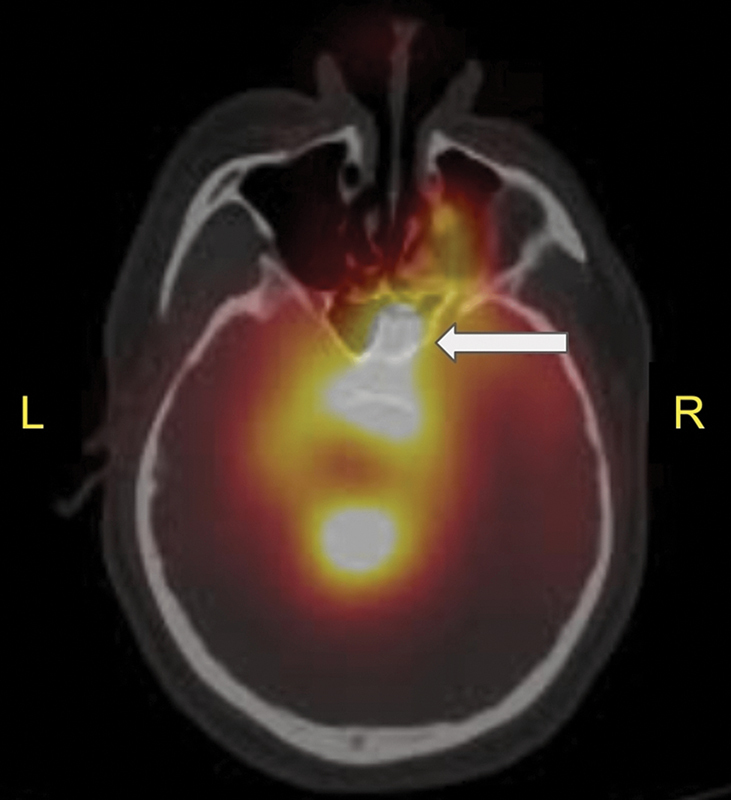
Cerebrospinal fluid leak into the right ethmoid and sphenoid air sinuses, and into the nasal cavity ( *solid white arrow* ).

A 41-year-old male patient presented with intermittent watery nasal discharge for 1 year, which could not be sniffed back. He was evaluated elsewhere, diagnosed with spontaneous CSF rhinorrhea, and underwent endoscopic epidural blood patch repair 4 months ago. He developed recurrent CSF rhinorrhea in the immediate postoperative period and was referred to our institution for further management. Comorbidities included poorly controlled diabetes mellitus, hypertension, and complex regional pain syndrome involving the right forearm and arm. He was referred to the Department of Nuclear Medicine in our institution for radionuclide cisternography to locate the site of leak. On the day of the scan, ^99m^ Tc DTPA was freshly prepared using a predispensed sterile formulated kit under strict aseptic conditions. The kit contained 35 mg of DTPA and 2 mg of stannous chloride dihydrate in freeze-dried form. Sterile sodium pertechnetate was freshly eluted from a molybdenum-technetium generator under aseptic conditions and ^99m^ Tc DTPA was prepared by adding two milliliters containing 740 megabecquerels (MBq) or 20 millicurie (mCi) of sodium pertechnetate to the radiopharmaceutical kit after attaining room temperature. The reaction vial was then placed in a boiling water bath for 5 minutes and then cooled to attain room temperature. The tagged ^99m^ Tc DTPA was measured as 700 MBq (∼ 19 mCi) by the dose calibrator. Radiochemical purity was assessed by paper chromatography using saline and acetone as solvents and Whatman filter paper strips III, and was found to be approximately 99%. Per protocol, he underwent lumbar puncture in the median portion of L4 to L5 level in a sitting position without imaging guidance. About 30 milliliters of CSF was drawn into three sterile containers for analysis following which 220 MBq (∼ 6 mCi) of ^99m^ Tc DTPA was injected intrathecally. The patient was instructed to lie down in the Trendelenburg position. Within 60 seconds, the patient developed a diffuse erythematous rash over the chest and shoulders, along with profuse sweating. He also complained of headache and watery discharge from the nose that could not be sniffed back. An intravenous access was obtained and the patient was administered 200 mg hydrocortisone immediately. The head end was elevated by 15 degrees. The patient then complained of pain in the lower limbs and numbness spreading to the level of the nipples (T4 dermatome). On examination, the patient appeared anxious; he had tachypnea but was able to communicate well. He was also hypertensive and had tachycardia. The lungs were clear on auscultation and saturation was maintained via high flow nasal cannula followed by room air after one hour. The erythematous rash and sweating were resolved within minutes of administration of intravenous cortisone. He was administered a bolus dose of midazolam and an infusion of dexmedetomidine. The patient was reassured and Merocel nasal packing of the right naris was performed one hour after injection. The patient was then shifted to the department of nuclear medicine for delayed static planar and single-photon emission computed tomography/computed tomography imaging. CSF leak into the right ethmoid and sphenoid air sinuses, and into the nasal cavity, was noted ( Fig. 1 ). No abnormal tracer distribution was noted at the site of injection that would suggest extravasation of the tracer. As the patient was stable and the procedure was complete, he was shifted back to the ward 6 hours after the tracer injection. A few hours later, it was observed that his peripheral oxygen saturation was in the range of 90 to 92% and he was tachypnic with a respiratory rate of 30 breaths per minute. He was shifted back to the intensive care unit and on evaluation he was found to have high blood pressure in the range of 170/100 mm Hg, decreased ejection fraction, and B lines on lung ultrasound. Auscultation revealed fine crackles. Accelerated hypertension-related cardiogenic pulmonary edema was suspected and he was managed with furosemide and noninvasive positive pressure ventilation. He was observed for 24 hours during which his blood pressure and ultrasound findings normalized. No other possible delayed reactions were noted during the rest of his hospital stay.

## Discussion

Frequency of drug reactions following the administration of radiopharmaceuticals is generally low and therefore, information regarding the same may be lacking in terms of crisp data. However, the uncommonly used routes of administration such as intrathecal route may warrant special attention due to the possibility of rare, but significant adverse hypersensitivity reactions. Most case reports highlight the occurrence of aseptic meningitis following radionuclide cisternography. 7 8 Vasomotor and skin reactions have been reported with the use of ^99m^ Tc DTPA, while its intrathecal administration has been reported to cause neurological symptoms including paraesthesias due to the formation of neurotoxic trisodium salts. 5 9 Animal studies conducted by Verbruggen et al in 1982 suggested that paralysis could be due to chelation of calcium and magnesium ions in the CSF by the trisodium salts, thus resulting in their depletion from the spinal cord. 6 In our patient, it was not possible to determine with certainty which component of the radiopharmaceutical preparation led to the development of an adverse allergic reaction. Additionally, it was not possible to establish a cause–effect relationship between administration of radiotracer and the delayed presentation of cardiogenic pulmonary edema. It is difficult to rule out the possibility of contribution by the patient's concurrent medical comorbidities and medications. No previous exposure sensitizing this patient to the contents of the radiopharmaceutical preparation could be traced. Radionuclide cisternography is not routinely performed in every nuclear medicine facility. Hence, when performed, it is crucial to be prepared for bizarre adverse drug reactions while administering ^99m^ Tc DTPA intrathecally. We recommend that the patient should have a patent intravenous cannula in situ prior to the procedure. Additionally, the injection should be performed with close monitoring of vitals, and resuscitation drugs and equipment close at hand. Patients should be monitored for 24 hours in the inpatient set-up whenever feasible.

## Conclusion

Herein, we report an interesting case of adverse allergic reaction to intrathecal administration of ^99m^ Tc DTPA who later developed cardiogenic pulmonary edema due to accelerated hypertension and was managed in the critical care unit.
